# Anatomical analysis of the abdominal aorta in a South African sample: influence of age and sex

**DOI:** 10.1007/s00276-024-03502-x

**Published:** 2024-10-21

**Authors:** Pheladi Mokoena, Robyn Lunn-Collier, Lee-Roy Witbooi, Karin Baatjes, Kerri Keet

**Affiliations:** 1 Department of Biomedical Sciences, Faculty of Medicine and Health Sciences, Division of Clinical Anatomy, Stellenbosch University, Cape Town, South Africa; 2 Division of Radiodiagnosis, Tygerberg Hospital, Cape Town, South Africa; 3 Faculty of Medicine and Health Sciences, Division of Surgery, Stellenbosch University, Cape Town, South Africa

**Keywords:** Abdominal aorta, Age, Anatomical variation, Computed tomography angiography, Endovascular treatment, Sex

## Abstract

**Purpose:**

The anatomy of the abdominal aorta (AA) varies with age and sex; however, limited studies exist from South Africa. Given the increased incidence of endovascular treatment of the AA, reference values are relevant for interventionalists for improving the safety of endovascular procedures. Therefore, the study aimed to determine the lengths, diameters and tortuosity of the AA and their association with age and sex in a South African sample.

**Methods:**

After ethical approval, 97 computed tomography angiography (CTA) scans from an adult sample (54 male and 43 female), mean age 48.5 ± 17.2 years were analysed. The aortic length was measured from the origin of the coeliac trunk to the bifurcation point of the AA. The lumen diameters of the aorta were measured at three landmarks. Tortuosity of the AA was quantified with the tortuosity index and its prevalence was determined.

**Results:**

The AA was longer in males and showed a significant weak positive correlation with age. The mean diameters of the AA were larger in males and had a significant strong positive correlation with age in both sexes (*p* < .001). There was a strong positive correlation between age and tortuosity in both sexes (*p* < .001). The prevalence of a tortuous c-shaped-curve phenotype was 8.2%, with a 7:1 male-to-female ratio.

**Conclusion:**

The dimensions and tortuosity differed between sexes and varied significantly with age. These findings may contribute towards reference values in the South African setting, inform patient selection and complement decision-making of endovascular treatment strategies.

## Introduction

The efficacy and safety of endovascular intervention of the abdominal aorta (AA) partly relies on knowledge of anatomical variation [4, 12]. Defining the range of variation of the AA is relevant for durable and patient-appropriate stent graft design [3] in endovascular aneurysm repair (EVAR) and for procedures traversing the AA such as transcatheter aortic valve replacement (TAVR) [14]. Preprocedural computed tomography angiography (CTA) images are essential in determining AA anatomy for the planning of endovascular interventions [14, 16].

Variation in the AA may be congenital or acquired and associated with demographic factors and cardiovascular risk factors [5, 8, 12, 24]. Due to its elastic properties, the length, diameter and tortuosity of the AA increases with age [8, 12, 22–24] and displays sexual dimorphism [1, 8, 22–24]. Tortuosity describes the presence of abnormal turns in a vessel [6]. However, the variation of the AA and the influence of age and sex have received little attention in research from South Africa, and the range of dimensions and tortuosity remains unknown. Thus, the aim of this study was to determine the length, diameter, tortuosity index and tortuosity prevalence of the AA in an adult South African sample using CTA. The influence of age and sex on AA anatomy were also analysed.

## Materials and methods

The study was retrospective, descriptive and cross-sectional in nature. Ethical approval was obtained (reference number: U22/04/168) and a waiver of consent was granted. Contrast-enhanced CTA scans were accessed on the Picture Archiving Communication System (PACS) database. Patients were scanned in a supine position with either a Phillips Ingenuity 128 CT scanner (Koninklije Phillips, Amsterdam, The Netherlands) with a slice thickness of 1 mm, or a CT-128 Somatom Definition Edge scanner (Siemens Healthineers AG, Forchheim, Germany) with a slice thickness of 0.75 mm. Intravenous contrast was injected (95 ml at 4 ml/s). An a priori sample size calculation indicated a sample size ranging from 174 to 268. The scans were stratified by biological sex and age into decade groups. The medical history of the patients including cardiovascular risk factors and disease status were unknown.

Scans obtained between 1 January 2014 and 31 August 2022 were included if they were clearly contrasted with all landmarks visible. Those with injury to the AA or visible signs of vascular diseases that are likely to affect vessel anatomy, namely aneurysms (diameter > 30 mm) [13] or severe atherosclerosis, were excluded.

The CTA images were analysed using Phillips IntelliSpace Portal system version 12.1 (Phillips, Medical Systems Technologies Ltd, 2020, Netherlands). The length, diameter and tortuosity of the AA were measured on two-dimensional (2D) and three-dimensional (3D) reconstructions using published landmarks [8, 12, 22, 24, 26]. The AA length was measured along the centerline from immediately superior to the coeliac artery to immediately superior to the aortic bifurcation. The maximum and minimum lumen diameters of the AA were measured at three landmarks perpendicular to the vessel’s centerline: immediately superior to the coeliac trunk, superior to the left renal artery and superior to the bifurcation point of the aorta. The average diameter at each landmark was calculated to account for irregularly shaped vessels.

Tortuosity was quantitatively assessed by calculating the tortuosity index (TI) from the ratio between the centerline length and the linear distance, which was the minimum distance measured between the two length landmarks. A TI value of 1.0 indicates a straight-line vessel, whereas larger values indicate a tortuous vessel [6, 28]. The TI indicates the severity of tortuosity, however not the appearance, therefore phenotypic descriptions (c-shaped or s-shaped curves, kinked, singular loops or coils) [9] were used to further describe the tortuosity.

Statistical analysis was completed using IBM Statistical Product and Service Solutions (SPSS) ^®^ Statistics (Version 28, IBM Corp., 2021Armonk, New York, United States). The Shapiro-Wilk test was used to assess the distribution of the data. The Student’s t-test for two independent groups was used to compare the length and diameter between sexes. The Pearson correlation coefficient analysed the relationship between length and diameter with age for each sex. Associations of age and sex with AA dimensions were further analysed in simple and multiple linear regression models. Analysis of variance (ANOVA) was used to compare dimension values between age groups. The Spearman rank order correlation determined the association of TI values with age, while the Mann-Whitney U test compared TI values between males and females. Intra- and inter-observer reliability was assessed using the intraclass correlation coefficient (ICC) on a randomly selected 10% subset of the sample. Confidence intervals for normality tests, percentages and statistical test coefficients were set at 95%. P- values less than or equal to 0.05 were considered statistically significant.

## Results

A total of 97 CTA scans were analysed after applying the inclusion and exclusion criteria. The scans were stratified into decade groups, ranging from 20 to 79 years (Table 1). The mean age of the males (*n* = 54) was 44.91 ± 17.51 (21–74 years) and the females (*n* = 43) was 53.05 ± 15.96 (23–79 years). The dimension values, excluding the TI values were normally distributed and the ICC coefficients indicated excellent intra- and interobserver reliability (range 0.96–1.00, *p* < .001).

**Table 1:** Sample characteristics of the final sample

Age group (Years)	20–29	30–39	40–49	50–59	60–69	70–79	Total
**Male**	13	11	7	7	9	7	54
**Female**	5	5	8	7	11	7	43
**Total**	18	16	15	14	20	14	97
**Mean age ± SD (Years)**	24.7 ± 2.5	33.1 ± 2.6	44.4 ± 3.0	55.7 ± 3.1	62.8 ± 3.0	73.5 ± 3.1	48.5 ± 17.2

The overall mean length of the AA was 125.87 ± 17.84 mm (range: 86.00–168.27 mm). The AA was longer in males (127.85 ± 19.33 mm, range: 86.00 –168.27 mm) than in females (123.37 ± 15.60 mm, range: 88.33 –152.00 mm) although this difference was not significant (*p* = .222). A weak positive correlation was found between the length and increasing age in both sexes. This correlation was statistically significant in males (*r* = .30, r^2^ = 0.09, *p* = .026), although not in females (*r* = .09, r^2^ = 0.01, *p* = .549). The length was significantly larger in the 70–79 year group than in the 20–29-year-olds (*p* = .017, Table 2). Simple linear regression indicated that age was significantly associated with AA length variability in males to a small degree (F (1,52) = 5.28, *p* = .026, R^2^ = 0.09). However, a combination of age and sex were significant positive predictors of length to a small degree by 6.6% according to multiple linear regression analysis (F (2, 94) = 3.32, *p* = .041, R^2^ = 0.07).

**Table 2:** Mean length of the abdominal aorta in males and females in six decadal groups

Years	Mean ± SD (mm)	
	Male	Female
20–29	120.31 ± 14.30	124.19 ± 12.03
30–39	125.93 ± 12.82	114.99 ± 10.69
40–49	130.22 ± 28.21	127.57 ± 9.76
50–59	121.65 ± 22.67	117.29 ± 14.83
60–69	127.66 ± 16.40	122.37 ± 20.92
70–79	148.92 ± 15.82	131.68 ± 16.37

**Fig. 1:**
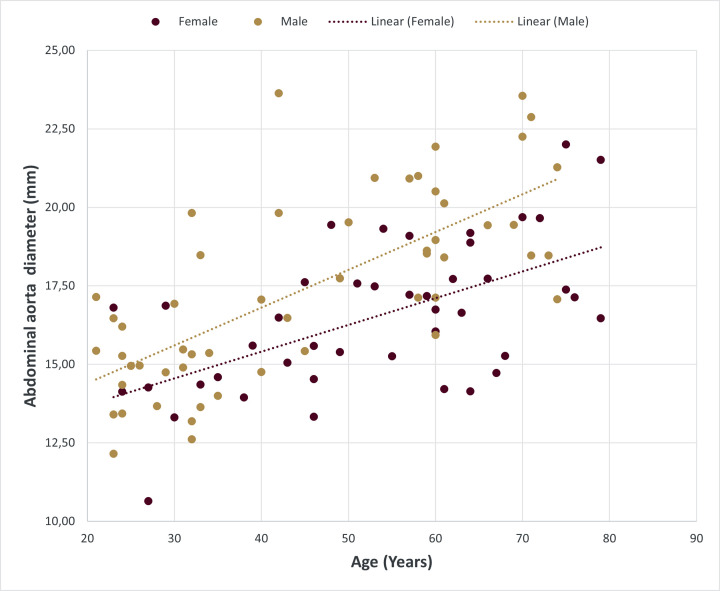
Scatterplot with a linear trendline showing the correlation between age and the abdominal aorta (AA) diameter in males (m: *r* = .71, *p* < .001) and females (f: *r* = .59, *p* < .001)

The overall mean diameter of the AA was 17.01 ± 2.72 mm (range: 10.64–23.63 mm). The mean diameters tapered inferiorly from the celiac trunk to the bifurcation point. The diameters were larger in males than in females at all measurement levels (Table 3), however the difference in mean diameter between sexes was not statistically significant (*p* = .115). There was a significant strong positive correlation between diameters and age in both males (*r* = .71, r^2^ = 0.50, *p* < .001) and in females (*r* = .59, r^2^ = 0.35, *p* < .001) (Fig. 1). Diameters were strongly associated with an increase in age in both sexes (*p* < .001). Multiple linear regression confirmed that age and sex are significant predictors of AA diameter (F (2, 94) = 39.18, *p* < .001, R^2^ = 0.46). Thus, 45.5% of the variability in AA diameters may be explained by a combination of age and sex.

**Table 3:** Mean diameters of the abdominal aorta (AA) at different measuring landmarks in the sample (*n* = 97)

Landmark	Mean ± SD (mm)	
	Male	Female
**Coeliac trunk**	20.00 ± 3.77	19.65 ± 2.89
**Left renal artery**	17.68 ± 2.98	16.65 ± 2.40
**Bifurcation point**	14.44 ± 2.69	13.25 ± 2.29

The AA diameters were larger in males than in females in all age groups (Table 4). There were statistically significant differences between the age groups within males (F (5, 480) = 12.16, *p* < .001) and within females (F (5, 37) = 8.93, *p* < .001). Interestingly, in males the mean AA diameter was smaller in the 70–79 age group in comparison to the 60–69 years age group. Generally, in males the AA diameters were significantly smaller in the 20–29 years age group than in the older age groups (*p* < .05). The AA diameters were significantly different between age groups that differed by two decades apart, for example, the diameter was smaller in the 30–39-year-olds in comparison with the 50–59 years age groups in both males and females (*p* < .05).

**Table 4:** Mean diameter of the abdominal aorta in males and females in six decadal groups

Age Group (years)	Mean ± SD (mm)	
	Male	Female
20–29	14.78 ± 1.39	14.54 ± 2.55
30–39	15.43 ± 2.22	14.36 ± 0.84
40–49	17.84 ± 3.04	15.93 ± 1.90
50–59	19.52 ± 1.51	17.59 ± 1.35
60–69	20.57 ± 2.53	16.48 ± 1.78
70–79	17.39 ± 2.97	16.52 ± 2.32

The median TI value for the AA was 1.01 ± 0.03 (range: 1.00–1.14). The TI values were greater in the 60–69 and 70–79 years age groups than in the younger age groups in both sexes (Table  5). There was a strong positive significant correlation between age and tortuosity in both males and females (r_s_ (52) = 0.60, *p* < .001 in males and r_s_ (41) = 0.60, *p* < .001 in females). Tortuosity severity was significantly greater in males than females (U = 835.00, *p* = .018).

**Table 5:** Tortuosity indices for the abdominal aorta in males and females amongst different age groups

Age groups	Median ± IQR (max) (mm)	
Years	Male	Female
20–29	1.01 ± 0.02 (1.02)	1.01 ± 0.01 (1.01)
30–39	1.00 ± 0.00 (1.01)	1.01 ± 0.01 (1.02)
40–49	1.01 ± 0.01 (1.10)	1.01 ± 0.00 (1.02)
50–59	1.01 ± 0.02 (1.04)	1.01 ± 0.01 (1.02)
60–69	1.01 ± 0.01 (1.09)	1.02 ± 0.02 (1.04)
70–79	1.05 ± 0.05 (1.14)	1.06 ± 0.09 (1.12)

**Fig. 2:**
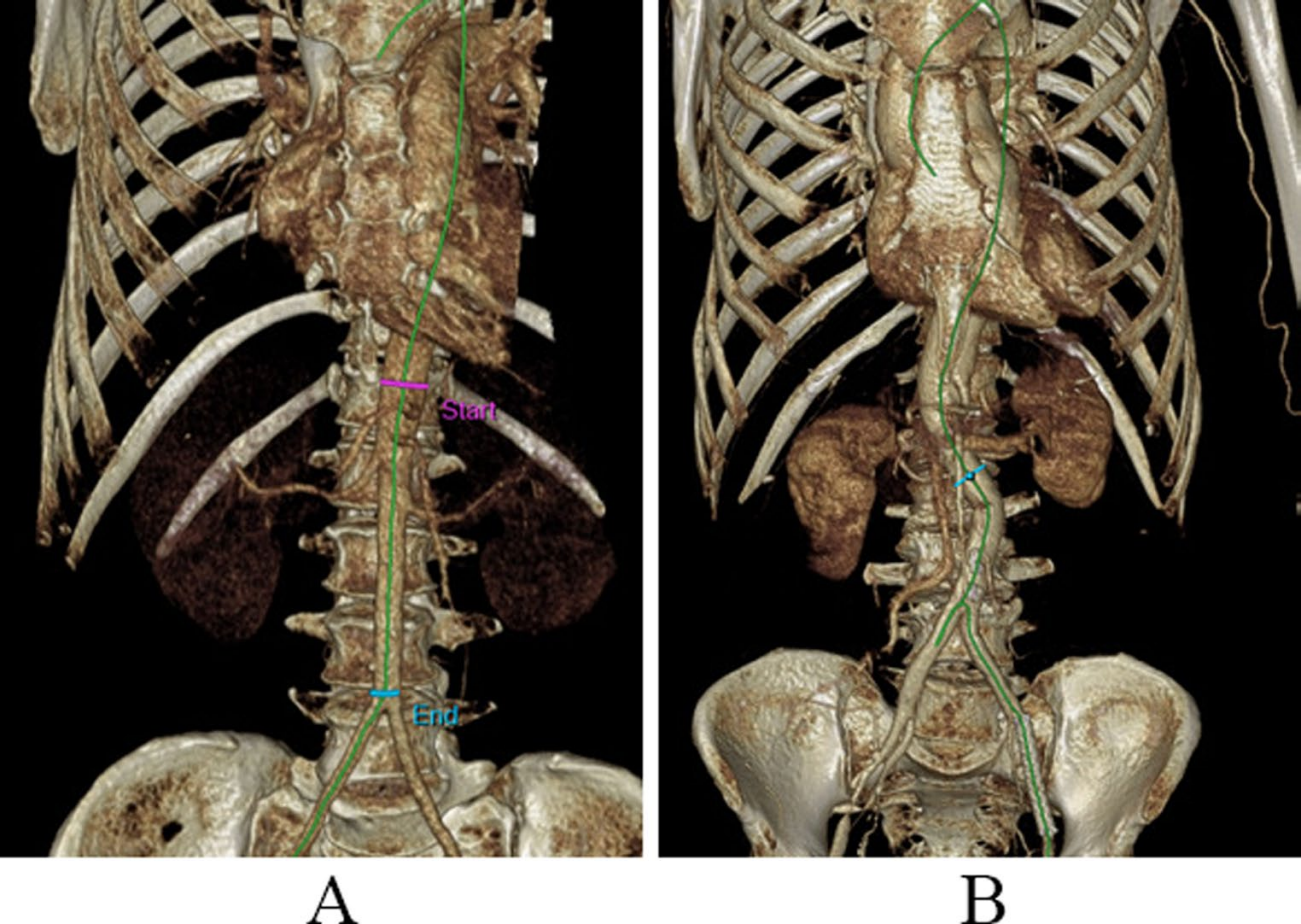
Computed tomography angiography scans showing straight (**a**) and C – shaped (**b**) tortuosity of the abdominal aorta

The prevalence of tortuous c-shaped curved phenotypes was 8.25% with a 7:1 male-to-female ratio. The c-shaped tortuous phenotype (Fig. 2) was observed in patients older than 42 years. No other tortuosity phenotypes were observed.

## Discussion

This study determined the morphometrics of the AA in different adult age groups in a South African sample of both sexes. The length, diameter, tortuosity severity and tortuosity prevalence varied between sexes and increased with age. The lengths and diameters were larger in males than in females, although the differences between sexes were not statistically significant. The length and diameters increased with age. The impact of age was bigger on diameter than on length.

The mean length was 127.85 ± 19.33 mm in males and 123.37 ± 15.63 mm in females. The length reported by Rylski et *al.* [24] (contrast-enhanced CT, *n* = 195, age range 20 to 96 years), was longer in North American adults than in the present study for males (144.00 ± 25 mm vs. 128.17 ± 19.33 mm) and for females (128 ± 20 mm vs. 121.00 ± 15.63 mm). In previous studies, larger lengths were significantly associated with male sex, however after adjusting for differences in body size, the differences between sexes were no longer significant [1, 24]. The length increased with age in both males and females, however the weak positive association length to age was statistically significant in males only. Rylski et al. [24] reported that age was significantly associated with increased lengths that were indexed to body surface area (BSA) in both males and females. However, BSA indexed values were not assessed in the study.

Previous studies have determined the AA diameter using different imaging analysis protocols and samples [8, 22, 23]. The measurement landmarks in the present study are however comparable with other studies that used multidetector CT [22, 23] and ultrasonography [8]. The AA tapered inferiorly towards the bifurcation point by 30.03% (at a ratio of 1.43. The maximum lumen diameter values proximal to the coeliac trunk were slightly smaller in the present study (21.21 ± 3.83 mm in males and 20.69 ± 2.97 mm in females) when compared to a Danish study (23.0 ± 2 mm in males and 21.2 ± 2 mm in females [22] of a large sample size (*n* = 902, median age 52 years). The mean diameter superior to the left renal artery in the present study was 17.7 ± 3.0 mm in males and 16.7 ± 2.4 mm in females. At this level, the average of the anteroposterior (AP) and transverse diameter (TD) values (AP: 15.57 ± 1.34 mm, TD: 17.10 ± 1.31 mm) reported for both sexes by Gameraddin [8] in a Sudanese sample (*n* = 110, mean age 43 ± 8 years) were smaller than the mean value (17.23 ± 2.77 mm) reported in the present study. However, Gameraddin [8] did not report the diameters for the sexes separately. At the bifurcation point, the mean outer wall diameters were larger in a North American study (*n* = 3432, mean age 52 ± 10 years) (18.7 ± 2.7 mm in males and 16.0 ± 1.9 mm in females) [23] than the mean lumen diameters in the present study (14.4 ± 2.7 mm in males and 13.9 ± 2.6 mm in females). The inclusion of wall thickness (outer to outer wall diameters) has however been suggested to increase diameter values by approximately 4 mm [17]. The diameter reported in the Sudanese study [8] at the AA bifurcation point (AP: 13.47 ± 1.23 mm, TD: 15.38 ± 2.13 mm) were similar to the present study (13.91 ± 2.58 mm). Thus, the diameters reported by these specific studies [22, 23] were larger than the present study, while those of another study [8] from the African continent were similar.

This study confirms findings from previous studies that age and sex influence the AA diameter. According to Kamenskiy et al. [12], age is the most important contributor to variation in diameter, followed by sex and BSA. Furthermore, Kamenskiy et al. reported a smaller effect of cardiovascular risk factors on AA anatomy than that of demographics [12]. Similar to previous studies [8, 22], statistically significant differences in diameter were not found between sexes, however there was a significant positive association between increasing age and diameter in both males and females at all landmarks. Results from the present study support Rylski et al.’s [24] statement that AA diameters vary significantly with each life decade. Furthermore, the positive correlation between age and diameter was strongest in males, with the the difference in diameter between male and females greatest in the 60–69-year age group, suggesting that the diameter increases at a higher rate in older males [29]. Wolak et al. [29] attributed this greater mean diameter difference between older male and female age groups to the combined interactions of BSA, smoking, hypertension and dyslipidaemia.

The TI index is commonly used to measure tortuosity as it has been reported to be reliable [25]. In support of this, the intra- and inter reliability of the TI index values were high in this study. Tortuosity of the AA increased significantly (*p* < .001) with age in both males and females, which agrees with previous findings [3, 12]. The TI values were significantly higher in males than females (*p* = .018). In contrast, Belvroy et al. [3] and Tawfik et al. [25] reported no significant differences in tortuosity between males and females (*p* = .626 and *p* = .208, respectively). The prevalence of tortuous AA phenotypes was 8.25% and they were more common in males and associated with older age groups, which agrees with previous studies [21].

Endovascular treatment is the preferred method to open surgery for patients at high-risk for mortality and paraplegia [7], even in the presence of tortuous phenotypes [10, 15]. Low to middle-income countries, such as South Africa, mainly import their medical devices from high-income countries and there may be a mismatch between the design of these devices to the local patient anatomy and the economic setting [19]. Stent grafts have relatively fixed lengths and diameters, thus finding the best-fit stent graft for a patient may be challenging due to variability in AA anatomy with age and sex [12]. Furthermore, tortuosity of the AA in older age groups may complicate endovascular treatment by increasing the risk of arterial perforation and rupture during sheath and valve introduction [14], leading to stent graft migration or increase the risk of endoleaks [3]. Thus, results from this study may inform endovascular design for the South African adult population and contribute to the guidelines for management of aneurysmal disease [27]. In addition, this study provides reference values of the range of dimensions and tortuosity in a sample with minimal or no vascular disease. As the AA anatomy varies with age and sex, separate reference values for both sexes in each age decade are important for evaluation of dilations or aneurysms of the AA [2, 18, 20].

The imaging modality and type of diameter reported is essential for the diagnosis of abdominal aortic aneurysms (AAA). Two studies have recommended the use of outer wall-to-outer wall diameters in the diagnosis of aneurysms. Using ultrasonography, one study demonstrated that lumen diameters or inner-to-inner wall (lumen) diameters may lower the threshold values for screening of aneurysms [17]. Furthermore, van Hout et al. [26] stated that outer wall-to-outer wall diameters are relevant for the diagnosis of aneurysms and surgical or transarterial intervention planning. Thus, future studies should establish standardized measurement guidelines [26] for the AA using different imaging analysis protocols to enable effective disease management and comparability between studies.

This study was conducted at a single centre thus limiting transferability of the results. The retrospective study design could investigate association between sex and age on the variation of the AA and not causation. Furthermore, a relatively small sample size of CTA scans were assessed with unequal age and sex stratification, thus results cannot be generalized. However, although the sample size was smaller than that which was required for statistical power according to sample size calculation, the findings are consistent with those of larger studies. Patient characteristics that may influence aortic vessel anatomy such as body mass index (BMI), cardiovascular risk factors and disease condition [12, 24, 29] were not assessed in this study as this information was not available to the researcher. There were some limitations present in the image analysis protocol. The aortic hiatus was not clearly visible on the imaging software, thus the researchers opted to use the coeliac trunk as the proximal landmark for AA length and diameter measurements. The coeliac trunk may not have originated from the same location in each individual. Additionally, some patient scans in the older age groups that showed visible signs of mild atherosclerosis were included in the sample because calcification is commonly present after the sixth decade of life [11].

## Conclusion

Age and sex had a significant influence on AA anatomy in this study. The length and lumen diameters increased with age and were larger in males than in females. Tortuosity was significantly associated with increasing age in both sexes and was more prevalent in males than females. This data contributes towards reference values for both sexes of each decade of age that may be useful in a South African setting. The findings from this study may inform interventionalists about patient selection and preprocedural planning for endovascular treatment, defining abdominal aortic disease thresholds and improved device design. Future studies should analyse larger samples with equal distribution between the different age groups and include measurements of outer wall-to-outer wall AA diameters.

## Data Availability

The data that support the findings of this study are available from the authors, but restrictions apply to the availability of these data, which were used under license from Stellenbosch University for the current study, and so are not publicly available. Data are, however, available from the authors upon reasonable request and with permission from Stellenbosch University. The data management is performed in accordance with SU data management policies and will be destroyed after a period of five years (2027).
